# Supplementary material for: Geothermal Gases Shape the Microbial Community of the Volcanic Soil of Pantelleria, Italy
Source: mSystems. 2020 Nov 3;5(6):e00517-20. doi: 10.1128/mSystems.00517-20 (PMC7646524; doi:10.1128/mSystems.00517-20)
Supplement: TABLE S1 [file mSystems.00517-20-st001.pdf]

Table S1. Relative abundance of the microbial community of site FAV2 in Pantelleria Island based on 16S rRNA gene sequences retrieved from the metagenome. Values are expressed in % and results of both DNA extraction method (CTAB and Power Soil kit) are shown separately.

	**1-10 cm**		**10-15 cm**		**15-20 cm**	
	CTAB	PS kit	CTAB	PS kit	CTAB	PS kit
Archaea	45	18	47	55	73	60
Bacteria	55	82	53	45	27	40
**Archaea**						
Crenarchaeota	0	0	0	4	0	0
Euryarchaeota	65	48	68	59	92	90
Thaumarchaeota	35	52	32	37	8	10
**Bacteria**						
Acidobacteria	4.8	7.7	0.0	0.0	2.4	15.6
Actinobacteria	14.3	20.7	6.7	26.6	1.5	22.2
Chloroflexi	31.4	40.9	36.6	31.5	11.2	24.3
Firmicutes	33.0	13.6	33.3	18.2	62.9	17.6
Proteobacteria	5.8	4.2	6.2	7.0	11.9	4.6
Verrucomicrobia	8.4	6.0	14.5	9.1	8.6	6.6
Other	2.3	6.8	2.7	7.6	1.6	9.2
**Chloroflexi**						
AKIW781	5.4	0.0	0.0	3.9	0.0	3.8
Ktedonobacteraceae	61.9	88.2	72.9	46.4	69.6	58.4
Thermobaculaceae	0.0	2.1	0.0	0.0	0.0	0.0
Uncultured	32.7	9.7	27.1	49.7	30.4	37.8
**Firmicutes**						
Bacillaceae	79.7	100.0	60.8	22.9	30.4	84.3
Family XVII	0.0	0.0	3.2	28.1	0.0	0.0
Paenibacillaceae	0.0	0.0	6.6	24.5	0.0	0.0
Thermoanaerobacteraceae	20.3	0.0	23.6	0.0	61.8	5.2
TSAC18	0.0	0.0	0.0	0.0	1.2	0.0
TTA-B61	0.0	0.0	0.0	0.0	1.0	0.0
Uncultured	0.0	0.0	5.8	24.5	5.6	10.5
**Proteobacteria**						
Burkholderiaceae	0.0	12.9	13.9	38.5	6.5	0.0
Methylococcaceae	0.0	36.6	38.7	0.0	0.0	100.0
Methylomonaceae	42.9	0.0	0.0	0.0	21.5	0.0
Rhizobiaceae	8.3	12.9	20.2	23.0	18.7	0.0
Sphingomonadaceae	48.8	37.6	0.0	38.5	16.1	0.0
Xanthomonadaceae	0.0	0.0	27.2	0.0	37.1	0.0
**Verrucomicrobia**						
Chthoniobacteraceae	14.7	19.2	8.4	18.8	0.0	58.2
Methylacidiphilaceae	85.3	80.8	91.6	81.2	100.0	41.8
