# Supplementary figures and images for: Geothermal Gases Shape the Microbial Community of the Volcanic Soil of Pantelleria, Italy
Source: mSystems. 2020 Nov 3;5(6):e00517-20. doi: 10.1128/mSystems.00517-20 (PMC7646524; doi:10.1128/mSystems.00517-20)

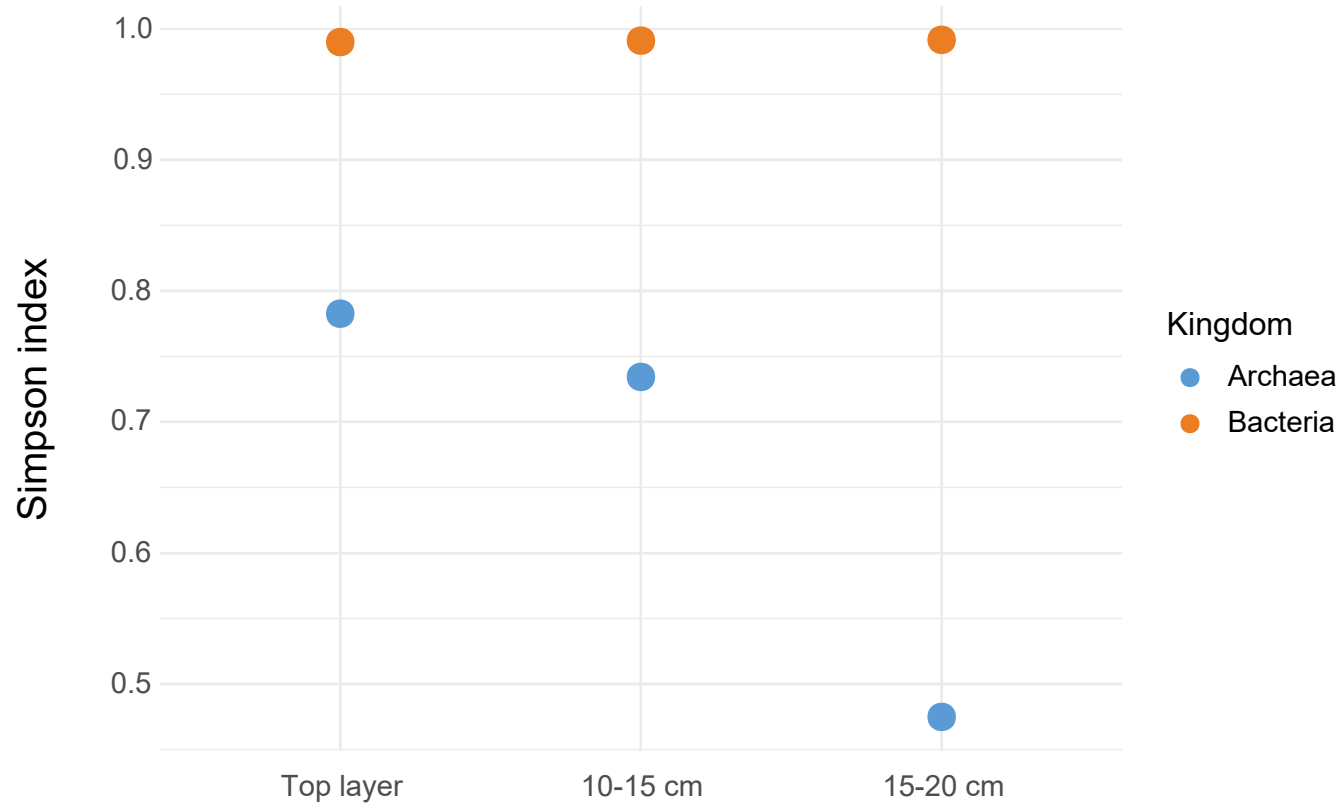

**Supplementary Fig. S1.**

Supplement: FIG S1 [file mSystems.00517-20-sf001.pdf]

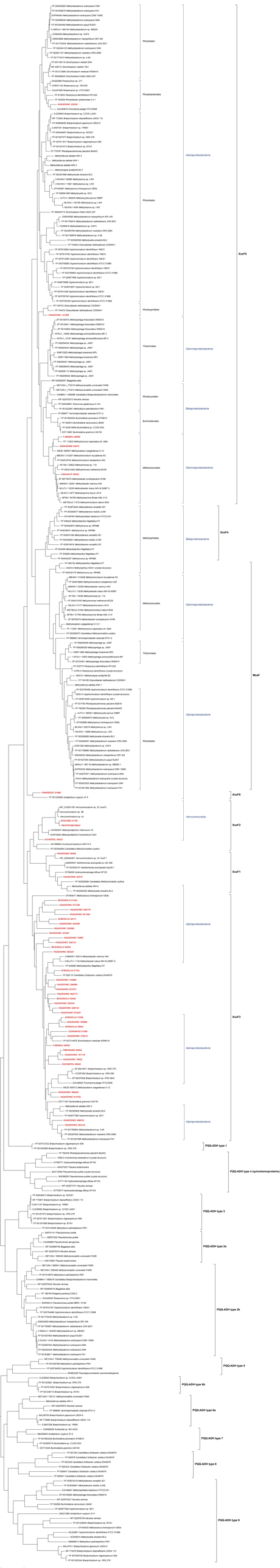

Supplement: FIG S2 [file mSystems.00517-20-sf002.pdf]
