# Supplementary material for: Geothermal Gases Shape the Microbial Community of the Volcanic Soil of Pantelleria, Italy
Source: mSystems. 2020 Nov 3;5(6):e00517-20. doi: 10.1128/mSystems.00517-20 (PMC7646524; doi:10.1128/mSystems.00517-20)
Supplement: TABLE S2 [file mSystems.00517-20-st002.pdf]

Table S2. List of HMM marker genes used for metagenomic analysis

sulfide dehydrogenase [flavocytochrome c] flavoprotein chain (EC:1.8.2.3)
cytochrome subunit of sulfide dehydrogenase
sulfide:quinone oxidoreductase [EC:1.8.5.4]
dissimilatory sulfite reductase alpha subunit [EC:1.8.99.5]
dissimilatory sulfite reductase beta subunit [EC:1.8.99.5]
anaerobic sulfite reductase subunit A
anaerobic sulfite reductase subunit B
anaerobic sulfite reductase subunit C
sulfur oxygenase/reductase [EC:1.13.11.55]
sulfur dioxygenase [EC:1.13.11.18]
sulfur-oxidizing protein SoxA; L-cysteine S-thiosulfotransferase [EC:2.8.5.2]
sulfur-oxidizing protein SoxB; S-sulfosulfanyl-L-cysteine sulfohydrolase [EC:3.1.6.20]
sulfane dehydrogenase subunit SoxC;
sulfur-oxidizing protein SoxY
S-disulfanyl-L-cysteine oxidoreductase SoxD [EC:1.8.2.6]
L-cysteine S-thiosulfotransferase [EC:2.8.5.2]
sulfur-oxidizing protein SoxZ
adenylylsulfate reductase, subunit A [EC:1.8.99.2]
adenylylsulfate reductase, subunit B [EC:1.8.99.2]
sulfate adenylyltransferase [EC:2.7.7.4]
sulfate adenylyltransferase subunit 2 [EC:2.7.7.4]
3'-phosphoadenosine 5'-phosphosulfate synthase [EC:2.7.7.4 2.7.1.25]
adenylylsulfate kinase [EC:2.7.1.25]
sulfate adenylyltransferase subunit 1 [EC:2.7.7.4]
bifunctional enzyme CysN/CysC [EC:2.7.7.4 2.7.1.25]
phosphoadenosine phosphosulfate reductase [EC:1.8.4.8 1.8.4.10]
sulfite reductase (NADPH) flavoprotein alpha-component [EC:1.8.1.2]
sulfite reductase (NADPH) hemoprotein beta-component [EC:1.8.1.2]
sulfite reductase (ferredoxin) [EC:1.8.7.1]
sulfur reductase molybdopterin subunit
sulfur reductase FeS subunit
sulfur reductase membrane anchor
thiosulfate reductase / polysulfide reductase chain A [EC:1.8.5.5]
thiosulfate reductase electron transport protein
thiosulfate reductase cytochrome b subunit
sulfite dehydrogenase (cytochrome C) [EC:1.8.2.1]
sulfite dehydrogenase (cytochrome C) subunit B
sulfite oxidase
sulfite dehydrogenase (quinone) subunit SoeA
sulfite dehydrogenase (quinone) subunit SoeB
sulfite dehydrogenase (quinone) subunit SoeC

Fe-Fe hydrogenase Group A1
Fe-Fe hydrogenase Group A2
Fe-Fe hydrogenase Group A3
Fe-Fe hydrogenase Group A4
Fe-Fe hydrogenase Group B
Fe-Fe hydrogenase Group C1
Fe-Fe hydrogenase Group C2
Fe-Fe hydrogenase Group C3
Fe-Fe hydrogenase Group Fe

Ni-Fe hydrogenase Group 1a
Ni-Fe hydrogenase Group Ib
Ni-Fe hydrogenase Group Ic
Ni-Fe hydrogenase Group Id
Ni-Fe hydrogenase Group Ie
Ni-Fe hydrogenase Group If
Ni-Fe hydrogenase Group Ig
Ni-Fe hydrogenase Group Ih
Ni-Fe hydrogenase Group Ii
Ni-Fe hydrogenase Group Ij
Ni-Fe hydrogenase Group Ik
Ni-Fe hydrogenase Group IIa
Ni-Fe hydrogenase Group IIb
Ni-Fe hydrogenase Group IIc
Ni-Fe hydrogenase Group IId
Ni-Fe hydrogenase Group IIe
Ni-Fe hydrogenase Group IIIa
Ni-Fe hydrogenase Group IIIb
Ni-Fe hydrogenase Group IIIc
Ni-Fe hydrogenase Group IIId
Ni-Fe hydrogenase Group IVa
Ni-Fe hydrogenase Group IVb
Ni-Fe hydrogenase Group IVc
Ni-Fe hydrogenase Group IVd
Ni-Fe hydrogenase Group IVe
Ni-Fe hydrogenase Group IVf
Ni-Fe hydrogenase Group IVg
Ni-Fe hydrogenase Group IVh
Ni-Fe hydrogenase Group IVi

methane/ammonia monooxygenase subunit A [EC:1.14.18.3 1.14.99.39]
methane/ammonia monooxygenase subunit B
methane/ammonia monooxygenase subunit C
methane monooxygenase component A alpha chain
methane monooxygenase component A beta chain
methane monooxygenase component A gamma chain
methane monooxygenase regulatory protein B
methane monooxygenase component D
methyl-coenzyme M reductase alpha subunit [EC:2.8.4.1]
methyl-coenzyme M reductase beta subunit [EC:2.8.4.1]
methyl-coenzyme M reductase gamma subunit [EC:2.8.4.1]
methyl-coenzyme M reductase subunit C
methane/ammonia monooxygenase subunit A [EC:1.14.18.3 1.14.99.39]
nitrogenase iron-iron protein, alpha chain [EC:1.18.6.1]
nitrogenase iron-iron protein, beta chain [EC:1.18.6.1]
nitrogenase delta subunit [EC:1.18.6.1]
nitrogenase molybdenum-iron protein alpha chain [EC:1.18.6.1]
nitrogenase molybdenum-iron protein beta chain [EC:1.18.6.1]
nitrogenase vanadium-iron protein, alpha chain [EC:1.18.6.1]
nitrogenase vanadium-iron protein beta chain [EC:1.18.6.1]
nitrogenase vanadium-iron protein delta chain [EC:1.18.6.1]
nitrogenase iron protein NifH [EC:1.18.6.1]
nitrate reductase / nitrite oxidoreductase, alpha subunit [EC:1.7.5.1 1.7.99.-]

nitrate reductase / nitrite oxidoreductase, beta subunit [EC:1.7.5.1 1.7.99.-]
periplasmic nitrate reductase NapA [EC:1.7.99.-]
nitrate reductase cytochrome c-type subunit
nitrate reductase / nitrite oxidoreductase, alpha subunit [EC:1.7.5.1 1.7.99.-]
nitrate reductase / nitrite oxidoreductase, beta subunit [EC:1.7.5.1 1.7.99.-]
nitrate reductase gamma subunit
nitrate reductase (NAD(P)H) [EC:1.7.1.1 1.7.1.2 1.7.1.3]
ferredoxin-nitrate reductase [EC:1.7.7.2]
assimilatory nitrate reductase catalytic subunit [EC:1.7.99.-]
assimilatory nitrate reductase electron transfer subunit [EC:1.7.99.-]
cytochrome c nitrite reductase small subunit
ferredoxin-nitrite reductase [EC:1.7.7.1]
nitrite reductase (cytochrome c-552) [EC:1.7.2.2]
nitrite reductase (NAD(P)H) [EC:1.7.1.4]
cytochrome c nitrite reductase, NrfD subunit [EC:1.7.2.2]
nitrite reductase (NADH) large subunit [EC:1.7.1.15]
nitrite reductase (NADH) small subunit [EC:1.7.1.15]
nitrite reductase (NO-forming) [EC:1.7.2.1]
nitrite reductase (NO-forming) / hydroxylamine reductase [EC:1.7.2.1 1.7.99.1]
octaheme c-type cytochrome, tetrathionate reductase family [EC:1.7.2.2]
nitric oxide reductase subunit B [EC:1.7.2.5]
nitric oxide reductase subunit C
nitrous-oxide reductase [EC:1.7.2.4]
hydrazine oxidoreductase A hydroxylamine oxidoreductase hydrazine dehydrogenase [EC:1.7.2.8]
hydrazine synthase subunit A [EC 1.7.2.7]
hydrazine synthase subunit B [EC 1.7.2.7]
hydrazine synthase subunit C [EC:1.7.2.7]
hydroxylamine dehydrogenase [EC:1.7.2.6]
urease subunit alpha [EC:3.5.1.5]
urease subunit beta [EC:3.5.1.5]
urease subunit gamma [EC:3.5.1.5]
urease subunit beta/gamma [EC:3.5.1.5]
urease [EC:3.5.1.5]
cyanate lyase [EC:4.2.1.104]
nitrile hydratase subunit alpha [EC:4.2.1.84]
nitrile hydratase subunit beta [EC:4.2.1.84]

cytochrome c oxidase subunit I [EC:1.9.3.1]
cytochrome c oxidase subunit I,coxAC; cytochrome c oxidase subunit I+III
cytochrome c oxidase subunit II [EC:1.9.3.1]
cytochrome c oxidase cbb3-type subunit I [EC:1.9.3.1]
cytochrome c oxidase cbb3-type subunit II
cytochrome c oxidase cbb3-type subunit III
cytochrome o ubiquinol oxidase subunit II [EC:1.10.3.10]
cytochrome o ubiquinol oxidase subunit IV
heme o synthase [EC:2.5.1.141]
cytochrome bd ubiquinol oxidase subunit I [EC:1.10.3.14]
cytochrome bd ubiquinol oxidase subunit II [EC:1.10.3.14]
cytochrome aa3-600 menaquinol oxidase subunit II [EC:1.10.3.12]
cytochrome aa3-600 menaquinol oxidase subunit I [EC:1.10.3.12]
cytochrome aa3-600 menaquinol oxidase subunit III,qoxC

PPQ-depenent methanol dehydrogenase [EC:1.1.2.7]

PPQ-depenent methanol dehydrogenase [EC:1.1.2.7]
methanol dehydrogenase (cytochrome c) subunit 2
methanol dehydrogenase [EC:1.1.1.244]
NDMA-dependent methanol dehydrogenase EC 1.1.1.244
alcohol oxidase [EC:1.1.3.13]
methylamine dehydrogenase light chain [EC:1.4.9.1]
methylamine dehydrogenase heavy chain [EC:1.4.9.1]
[methyl-Co(III) methylamine-specific corrinoid protein]:coenzyme M methyltransferase [EC:2.1.1.247]
[methyl-Co(III) methanol-specific corrinoid protein]:coenzyme M methyltransferase [ec:2.1.1.246]
glutathione-independent formaldehyde dehydrogenase [EC:1.2.1.46]
S-formylglutathione hydrolase [EC:3.1.2.12]
S-(hydroxymethyl)glutathione dehydrogenase / alcohol dehydrogenase [EC:1.1.1.284 1.1.1.1]
S-(hydroxymethyl)mycothiol dehydrogenase [EC:1.1.1.306]
5,6,7,8-tetrahydromethanopterin hydro-lyase [EC:4.2.1.147]
formylmethanofuran--tetrahydromethanopterin N-formyltransferase [EC:2.3.1.101]
methenyltetrahydromethanopterin cyclohydrolase [EC:3.5.4.27]
formate dehydrogenase [EC:1.17.1.9]
formate dehydrogenase major subunit [EC:1.17.1.9]
formate dehydrogenase beta subunit [EC:1.17.1.9]
formate dehydrogenase iron-sulfur subunit
formate dehydrogenase subunit gamma
formate dehydrogenase subunit delta [EC:1.17.1.9]
formate dehydrogenase (coenzyme F420) beta subunit [EC:1.17.98.3 1.8.98.6]
formate dehydrogenase (coenzyme F420) alpha subunit [EC:1.17.98.3 1.8.98.6]
formate dehydrogenase (NADP+) alpha subunit [EC:1.17.1.10]
formate dehydrogenase (NADP+) beta subunit [EC:1.17.1.10]
aerobic carbon-monoxide dehydrogenase small subunit [EC:1.2.5.3]
aerobic carbon-monoxide dehydrogenase medium subunit [EC:1.2.5.3]
aerobic carbon-monoxide dehydrogenase large subunit [EC:1.2.5.3]
ribulose-bisphosphate carboxylase large chain [EC:4.1.1.39] Form I
ribulose-bisphosphate carboxylase large chain [EC:4.1.1.39] Form II
ribulose-bisphosphate carboxylase large chain [EC:4.1.1.39] Form II/III
ribulose-bisphosphate carboxylase large chain [EC:4.1.1.39] Form III
ribulose-bisphosphate carboxylase large chain [EC:4.1.1.39] Form IV
ribulose-bisphosphate carboxylase small chain [EC:4.1.1.39]
phosphoribulokinase [EC:2.7.1.19]
acetyl-CoA synthase [EC:2.3.1.169]
acetyl-CoA decarbonylase/synthase complex subunit beta [EC:2.3.1.-]
acetyl-CoA decarbonylase/synthase complex subunit alpha [EC:1.2.7.4]
acetyl-CoA decarbonylase/synthase complex subunit gamma [EC:2.1.1.245]
acetyl-CoA decarbonylase/synthase complex subunit epsilon
acetyl-CoA decarbonylase/synthase complex subunit delta [EC:2.1.1.245]
anaerobic carbon-monoxide dehydrogenase catalytic subunit [EC:1.2.7.4]
anaerobic carbon-monoxide dehydrogenase iron sulfur subunit
glycine hydroxymethyltransferase [EC:2.1.2.1]
malate-CoA ligase subunit beta [EC:6.2.1.9]
malate-CoA ligase subunit alpha [EC:6.2.1.9]
3-hexulose-6-phosphate synthase [EC:4.1.2.43]
bifunctional enzyme Fae/Hps [EC:4.2.1.147 4.1.2.43]
3-hexulose-6-phosphate synthase / 6-phospho-3-hexuloisomerase [EC:4.1.2.43 5.3.1.27]
pyruvate ferredoxin oxidoreductase alpha subunit [EC:1.2.7.1]
pyruvate ferredoxin oxidoreductase beta subunit [EC:1.2.7.1]
pyruvate ferredoxin oxidoreductase delta subunit [EC:1.2.7.1]

pyruvate ferredoxin oxidoreductase gamma subunit [EC:1.2.7.1]
pyruvate-ferredoxin/flavodoxin oxidoreductase [EC:1.2.7.1 1.2.7.-]
malyl-CoA/(S)-citramalyl-CoA lyase [ec:4.1.3.24,4.1.3.25]
succinate semialdehyde reductase (NADPH) [EC:1.1.1.-]
2-oxoglutarate/2-oxoacid ferredoxin oxidoreductase subunit alpha [EC:1.2.7.3 1.2.7.11]
2-oxoglutarate/2-oxoacid ferredoxin oxidoreductase subunit beta [EC:1.2.7.3 1.2.7.11]
2-oxoglutarate ferredoxin oxidoreductase subunit delta [EC:1.2.7.3]
2-oxoglutarate ferredoxin oxidoreductase subunit gamma [EC:1.2.7.3]
ATP-citrate lyase alpha-subunit [EC:2.3.3.8]
ATP-citrate lyase beta-subunit [EC:2.3.3.8]

2-haloacid dehalogenase [EC:3.8.1.2]
tetrachloroethene reductive dehalogenase catalytic subunit PceA [EC:1.21.99.5]
DMSO reductase family type II enzyme, molybdopterin subunit / Perchlorate reductase subunit alpha (EC:1.97.1.-)
selenate/chlorate reductase subunit alpha ; clrA, serA [EC:1.97.1.9 1.97.1.1]
DMSO reductase family type II enzyme, iron-sulfur subunit / Perchlorate reductase subunit beta
clrb, serB; selenate/chlorate reductase subunit beta [EC:1.97.1.9 1.97.1.1]
serC, clrC; selenate/chlorate reductase subunit gamma [ec:1.97.1.9,1.97.1.1]
chlorite dismutase [EC:1.13.11.49]
arsenite oxidase small subunit [EC:1.20.2.1 1.20.9.1]
arsenite oxidase large subunit [EC:1.20.2.1 1.20.9.1]
arsenate reductase (glutaredoxin) [EC:1.20.4.1]
arsenate reductase (thioredoxin) [EC.1.20.4.- 3.1.3.48]
arsenate reductase, glutathione/glutaredoxin type
putative selenate reductase FAD-binding subunit
probable selenate reductase, molybdenum-binding subunit
putative selenate reductase [EC:1.97.1.9]
decaheme c-type cytochrome, OmcA/MtrC family
decaheme-associated outer membrane protein, MtrB/PioB family
