# Supplementary material for: Geothermal Gases Shape the Microbial Community of the Volcanic Soil of Pantelleria, Italy
Source: mSystems. 2020 Nov 3;5(6):e00517-20. doi: 10.1128/mSystems.00517-20 (PMC7646524; doi:10.1128/mSystems.00517-20)
Supplement: TABLE S3 [file mSystems.00517-20-st003.pdf]

Supplementary Table S3: Accession numbers of MAGs.

Number	Assembly accession	Taxonomy
1	GCA_902810565	Methanocella conradii
2	GCA_902806695	Methylobacter sp.
3	GCA_902810635	Chloroflexi sp.
4	GCA_902810765	Dehalococcoidetes sp. B11
5	GCA_902810725	Verrucomicrobiaceae sp.
6	GCA_902812485	Uncultured Archaeon
7	GCA_902810745	Dehalococcoidetes sp
8	GCA_902810705	Methylococcus sp.
9	GCA_902810735	Verrucomicrobiaceae sp.
10	GCA_902810715	Uncultured Bacterium
11	GCA_902810755	Dehalococcoidetes sp.
12	GCA_902810695	Rubrobacter sp.
13	GCA_902812495	Uncultured Bacterium
14	GCA_902812345	Chloroflexi sp.
15	GCA_902812405	Uncultured Bacterium
16	GCA_902812445	Methylococcus sp.
17	GCA_902812365	Acidobacterium sp.
18	GCA_902812395	Uncultured Bacterium
19	GCA_902812415	Acidobacterium sp.
20	GCA_902812455	Dehalococcoidetes sp.
21	GCA_902812465	Acidobacteria sp.
22	GCA_902812335	Chloroflexi sp.
23	GCA_902812355	Actinobacterium sp.
24	GCA_902812475	Actinobacterium sp.
25	GCA_902812385	Clostridia sp.
26	GCA_902812505	Uncultured Bacterium
27	GCA_902812435	Clostridia sp.
28	GCA_902812425	Rubrobacter sp
29	GCA_902812375	Uncultured Bacterium
30	GCA_902812875	Clostridiales sp.
